# The role of social support in the association between quality of life and demoralization in patients with inflammatory bowel disease: a cross-sectional study

**DOI:** 10.3389/fpsyg.2025.1689939

**Published:** 2025-12-02

**Authors:** Jingrui Ji, Haoran Gu, Sifei Lei, Pan Shen, Jie Zheng, Yinling Zhang

**Affiliations:** 1 Nursing Department, Air Force Medical University, Xi'an, China; 2 Traumatic Orthopedics, Army Hospital, Kashgar, Xinjiang, China; 3 Nursing Department, The PLA 77 Group Army Hospital, Leshan, Sichuan, China; 4 TCM Rehabilitation Department, Army 947 Hospital, Kashgar, Xinjiang, China; 5 Nursing Department, No. 980 Hospital of PLA Joint Logistics Support Force, Shijiazhuang, Hebei, China

**Keywords:** inflammatory bowel disease, demoralization, social support, quality of life, mediating effect

## Abstract

**Purpose:**

This study aims to investigate the associations among social support, quality of life, and demoralization in patients with inflammatory bowel disease (IBD), and to test the hypothesized mediating role of social support between quality of life and demoralization.

**Methods:**

A cross-sectional study was conducted using a convenience sampling approach. A total of 293 patients with inflammatory bowel disease (IBD) were recruited from the gastroenterology departments of three tertiary-level hospitals in Xi’an, China, between January and July 2024. Data were collected using a general information questionnaire, the Chinese version of the Demoralization Scale-II (DS-II), the Social Support Rating Scale (SSRS), and the Inflammatory Bowel Disease Questionnaire (IBDQ). Internal consistency reliabilities (Cronbach’s *α*) were 0.801, 0.825, and 0.986 for the DS-II, SSRS, and IBDQ, respectively. Correlation analyses and structural equation modeling (SEM) with bootstrapping (5,000 resamples, bias-corrected) were used to examine relationships and test mediation.

**Results:**

Social support was positively correlated with quality of life (*r* = 0.171, 95% CI [0.052, 0.285], *p* < 0.01), and negatively correlated with demoralization (*r* = −0.402, 95% CI [−0.499, −0.295], *p* < 0.01). Quality of life was negatively correlated with demoralization (*r* = −0.490, 95% CI [−0.575, −0.395], *p* < 0.01). Social support partially mediated the relationship between quality of life and demoralization, accounting for 22.12% of the total effect (indirect effect = −0.125, 95% CI [−0.178, −0.082]).

**Conclusion:**

Social support plays a significant partial mediating role in the relationship between quality of life and demoralization in IBD patients. These cross-sectional findings suggest that interventions aimed at enhancing social support may help mitigate demoralization and improve quality of life, though longitudinal or experimental studies are needed to confirm causal pathways.

## 1 Introduction

Inflammatory bowel disease (IBD), encompassing Crohn’s disease and ulcerative colitis, is a group of chronic, relapsing–remitting gastrointestinal disorders that impose a substantial burden on patients’ lives (Bisgaard et al., 2022). Beyond significant physical symptoms and complications, IBD profoundly impacts psychosocial well-being, often leading to diminished quality of life—a critical patient-reported outcome and a primary therapeutic goal. The incidence of IBD has been rising globally (Ng et al., 2017), with China now having the highest number of cases in Asia; forecasts suggest the number of IBD patients in China will reach 1.5 million by 2025 (Xu et al., 2023; Zhang, 2020).

A key psychological correlate in this population is demoralization, characterized by feelings of helplessness, hopelessness, loss of meaning, and impaired coping capacity (Frank, 1974). Unlike depression, which emphasizes affective and neurovegetative symptoms, demoralization reflects a subjective inability to cope and loss of purpose, often arising in response to persistent stress or illness (Robinson et al., 2016). This state of existential distress is increasingly recognized as a significant contributor to poor quality of life in chronic illness. Conversely, social support—derived from family, friends, and healthcare providers—represents a crucial psychosocial resource that may buffer stress, foster adaptive coping, and enhance resilience (Zhang, 2020).

Theoretical frameworks such as the Conservation of Resources theory (Hobfoll, 1989) and the Stress-Buffering Model (Cohen and Wills, 1985) posit that social support can mitigate the impact of stressors on mental health. While negative associations between demoralization and quality of life in IBD are established, and the protective role of social support is acknowledged, the mediating mechanisms linking these constructs remain insufficiently elucidated. Specifically, whether social support attenuates the link between demoralization and quality of life in IBD patients is not well understood.

**Figure 1:**
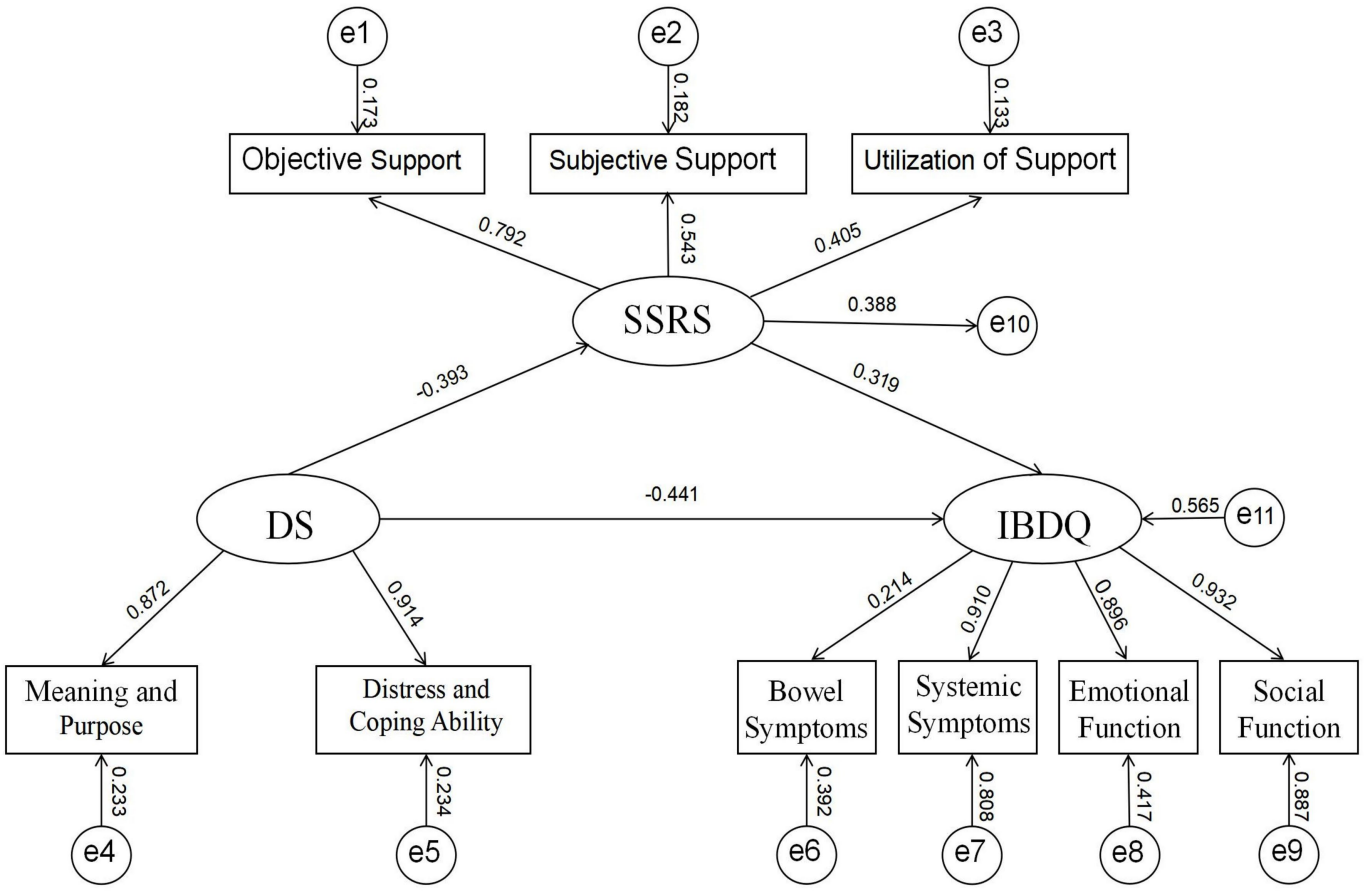
The mediating effect model of social support.

Therefore, this study aims to investigate the associations among quality of life, social support, and demoralization in IBD patients, and to test the hypothesized mediating role of social support between quality of life and demoralization. We propose a theoretical model (Figure 1) in which higher quality of life is associated with greater social support, which in turn is associated with lower demoralization. The findings may inform the development of targeted psychosocial interventions for this population.

## 2 Materials and methods

### 2.1 Study design and participants

This was a cross-sectional observational study. A convenience sample of 293 patients with IBD was recruited from the gastroenterology departments of three tertiary hospitals in Xi’an, China, between January and July 2024. Participants included 107 patients in the active phase and 186 in clinical remission.

### 2.2 Inclusion criteria

(1) Aged ≥18 years;
(2) Diagnosed with IBD according to the Chinese Consensus on Diagnosis and Management of Inflammatory Bowel Disease (Beijing) (Wu et al., 2018);
(3) Cognitively intact with normal verbal communication abilities;
(4) Provided written informed consent.

### 2.3 Exclusion criteria

(1) Comorbid primary diseases or life-threatening malignancies;
(2) Severe complications (e.g., intestinal obstruction, perforation, or colorectal cancer).

This study protocol was approved by the Ethics Committee of the First Affiliated Hospital of Air Force Medical University (also known as Xijing Hospital) (approval no.: KY20232129-C-1). Written informed consent was obtained from all participants prior to their enrollment in the study.

### 2.4 Sample size justification

Sample size was estimated using Monte Carlo power analysis for SEM with latent variables, based on anticipated small-to-medium effect sizes (*β* ≈ 0.20–0.30) for the mediation paths. With 5,000 replications and 80% power at *α* = 0.05, a minimum of 240 participants was required. Accounting for a 20% attrition rate, 293 participants were enrolled.

### 2.5 Measures

#### 2.5.1 Demographic and clinical characteristics questionnaire

Developed by the researchers, this tool collected data on age, gender, education, marital status, occupation, payment method, IBD duration, disease phase, and history of IBD-related surgery.

#### 2.5.2 Chinese version of demoralization scale-II (DS-II)

The 16-item DS-II (Robinson et al., 2016) was used in its validated Chinese version (Wu et al., 2021). It assesses two dimensions: Meaning and Purpose (8 items) and Distress and Coping Ability (8 items), rated on a 3-point Likert scale. Total scores range from 0 to 32, with higher scores indicating greater demoralization. Cronbach’s *α* in this study was 0.801.

#### 2.5.3 Social support rating scale (SSRS)

The 10-item SSRS (Xiao, 1994) assesses Subjective Support (4 items), Objective Support (3 items), and Utilization of Support (3 items). Total scores classify support as low (≤22), medium (23–44), or high (45–66). Cronbach’s α was 0.825 in this study.

#### 2.5.4 Inflammatory bowel disease questionnaire (IBDQ)

The 32-item IBDQ (Guyatt et al., 1989) was used in its Chinese version (Parkes et al., 2025) to assess Bowel Symptoms (10 items), Systemic Symptoms (5 items), Emotional Function (12 items), and Social Function (5 items). Items are scored 1–7; total scores range from 32 to 224, with higher scores indicating better quality of life. Cronbach’s *α* was 0.986.

### 2.6 Data collection

A total of 320 questionnaires were distributed. After excluding incomplete or invalid responses, 293 valid questionnaires were retained (effective response rate: 91.56%). Data were double-entered into Excel to ensure accuracy.

### 2.7 Data quality control

Common method variance was assessed using an unmeasured latent method factor approach, indicating average method variance of 18.3%, below the severe concern threshold. Missing data were minimal (mean rate = 1.2%) and handled using Full Information Maximum Likelihood (FIML) in SEM analyses, supported by sensitivity analyses showing robust results.

### 2.8 Statistical analysis

Data were analyzed using SPSS 26.0 and AMOS 27.0. Descriptive statistics summarized demographic and clinical characteristics. Pearson correlations examined relationships among key variables. Structural equation modeling (SEM) tested the mediating effect of social support. Model fit was assessed using *χ^2^/df*, CFI, TLI, GFI, and RMSEA. Bias-corrected bootstrap analysis (5,000 resamples) was used to test the significance of the indirect effect. Statistical significance was set at *p* < 0.05 (two-tailed). Assumptions of normality, linearity, and homoscedasticity were checked and met.

## 3 Results

### 3.1 Participant characteristics

A total of 293 IBD patients participated (mean age: 44.67 ± 12.93 years; range: 18–82). Other demographic and clinical characteristics are presented in Table 1.

**Table 1:** Baseline characteristics of IBD patients (*n* = 293).

Characteristic	Category	*N*	%
Sex	Male	164	56.0
	Female	129	44.0
Age (years)	18–44	145	49.5
	45–59	99	33.8
	60–74	42	14.3
	> 74	7	2.4
Education level	Primary school or below	19	6.5
	Junior high school	69	23.6
	High school/Vocational school	54	18.4
	College (associate degree)	59	20.1
	Undergraduate degree or above	92	31.4
Marital status	Married	247	84.3
	Unmarried	38	13.0
	Divorced	8	2.7
Employment status	Employed	127	43.3
	Retired	55	18.8
	Laid-off/Unemployed	15	5.1
	Other	96	32.8
Payment method	Medical insurance	178	60.8
	Self-pay	99	33.8
	Other	16	5.4
Monthly income (CNY)	3,000¥	106	36.2
	3,001~5,000¥	73	24.9
	>5,000¥	114	38.9
Disease type	Ulcerative colitis (UC)	230	78.5
	Crohn’s disease (CD)	63	21.5
Disease phase	Catabasis	186	63.5
	Active stage	107	36.5
Disease duration (years)	<1	32	10.9
	1~10	164	56.0
	11~20	88	30.0
	>20	9	3.1
History of IBD-related Surgery	Yes	55	18.8
	No	238	81.2

### 3.2 Quality of life, social support, and demoralization scores

The mean total IBDQ score was 167.01 ± 37.776, indicating a moderate quality of life. The mean SSRS score was 24.23 ± 5.54, indicating moderate-to-low social support. The mean DS-II score was 11.13 ± 6.71, indicating moderate demoralization; 127 patients (43.34%) scored in the moderate range (Wu et al., 2021; Xiao, 1994; Guyatt et al., 1989; Parkes et al., 2025; Byron et al., 2020; Fu et al., 2020; Woźniewicz and Cosci, 2023; Zhu et al., 2021; Vehling and Kissane, 2018; Liu et al., 2024). Dimension scores are shown in Table 2.

**Table 2:** Top 5 highest-scoring items on the demoralization scale in IBD patients (*n* = 293).

Items	Score	Min score	Actual score (mean ± SD)	Rank
12. I feel distressed about what has happened to me.	2	0	0.97 ± 0.58	1
8. I feel agitated.	2	0	0.93 ± 0.60	2
1. I feel I am hardly able to provide any value for others.	2	0	0.91 ± 0.59	3
10. I have many regrets about my life.	2	0	0.89 ± 0.59	4
11. I feel vulnerable.	2	0	0.83 ± 0.64	5

### 3.3 Correlations among key variables

Social support was positively correlated with quality of life (*r* = 0.171, 95% CI [0.052, 0.285], *p* < 0.01) and negatively correlated with demoralization (*r* = −0.402, 95% CI [−0.499, −0.295], *p* < 0.01). Quality of life was negatively correlated with demoralization (*r* = −0.490, 95% CI [−0.575, −0.395], *p* < 0.01) (see Table 3).

**Table 3:** Bivariate correlations among key variables (*n* = 293).

Variable	1	2	3
1. Demoralization	—		
2. Social support	−0.402**	—	
3. Quality of life	−0.490**	0.171**	—

**P < 0.01.

### 3.4 Mediating effect of social support

SEM results supported the hypothesized model (Figure 1). Fit indices were: *χ^2^/df* = 2.011, IFI = 0.918, CFI = 0.923, GFI = 0.914, RMSEA = 0.063 (90% CI [0.048, 0.078]), indicating good fit.

Path coefficients (Table 4) showed:

- Social support positively predicted quality of life (*β* = 0.319, *p* < 0.01)
- Demoralization negatively predicted quality of life (*β* = −0.441, *p* < 0.01)
- Social support negatively predicted demoralization (*β* = −0.393, *p* < 0.01)

**Table 4:** Path analysis results for the mediation model (*n* = 293).

Path	*β*	SE	*z*	*P*	95% CI
a: Demoralization → Social Support	−0.393	0.055	−7.15	<0.001	[−0.501, −0.285]
b: Social Support → Quality of Life	0.319	0.047	6.79	<0.001	[0.227, 0.411]
c’: Direct Effect (Demoralization → QoL)	−0.441	0.048	−9.19	<0.001	[−0.535, −0.347]
Indirect effect (a × b)	−0.125	0.024	—	—	[−0.178, −0.082]

The indirect effect was −0.125 (95% CI [−0.178, −0.082]), accounting for 22.12% of the total effect. Bootstrap results confirmed the significance of the mediation.

## 4 Discussion

### 4.1 Key findings and theoretical implications

This cross-sectional study confirms the intricate interrelationships among quality of life, social support, and demoralization in Chinese patients with IBD. Consistent with our hypothesis, social support was identified as a significant partial mediator in the relationship between quality of life and demoralization, accounting for 22.12% of the total effect. This finding illuminates a potential psychosocial mechanism, suggesting that the detrimental impact of impaired quality of life on a patient’s psychological state is not only direct but also operates indirectly by eroding the very fabric of their social support system. This aligns with the Conservation of Resources (COR) theory (Hobfoll, 1989), where quality of life can be viewed as a key personal resource. The loss of this resource (poor QoL) may hinder an individual’s capacity to invest in and maintain other critical resources, such as robust social networks, thereby increasing vulnerability to demoralization—a state characterized by resource depletion and an inability to cope effectively.

### 4.2 The plight of social support and its clinical relevance

The overall level of social support in our cohort was moderate-to-low, with notably deficient scores in the domains of objective support and support utilization. This disparity suggests that patients may perceive available support in theory but face tangible barriers in accessing practical help or are reluctant to actively seek it, potentially due to disease-related stigma or a desire to avoid being a burden (Byron et al., 2020; Fu et al., 2020). This deficiency in actionable support creates a critical intervention point. Our mediation model implies that enhancing tangible social support—such as facilitating access to community services, providing clear information on healthcare navigation, and counseling families on effective support strategies—could disrupt the pathway to demoralization. This approach moves beyond mere emotional reassurance to provide practical resources that bolster a patient’s sense of control and self-efficacy.

### 4.3 Demoralization in the context of chronic illness

The observed level of demoralization in our sample underscores its clinical significance in IBD, a finding consistent with research in other chronic and life-threatening conditions (Robinson et al., 2016; Zhu et al., 2021). Unlike depression, which is often the focus of clinical attention, demoralization encapsulates the profound existential crisis and loss of purpose that can accompany a relentless, unpredictable disease like IBD. The comparable scores in the Distress and Coping Ability and Meaning and Purpose dimensions reveal that patients are not only struggling with immediate emotional distress but are also grappling with a fractured sense of life meaning and future trajectory (Vehling and Kissane, 2018). This highlights the necessity of integrating meaning-centered interventions or dignity therapy into standard IBD care, aiming to help patients reconstruct a sense of purpose and coherence in life despite their illness.

### 4.4 The vicious cycle of QoL, social function, and demoralization

The strong negative correlation between demoralization and quality of life, particularly with the emotional and social function domains of the IBDQ, points to a potential vicious cycle. Demoralization can lead to social withdrawal and isolation, which in turn diminishes opportunities for positive social interactions and support, further exacerbating feelings of meaninglessness and hopelessness (Liu et al., 2024). Conversely, impairments in social function due to debilitating symptoms (e.g., urgency, fatigue) can directly fuel demoralization by preventing participation in valued life roles and activities (Byron et al., 2020). Therefore, interventions must be multifaceted, targeting both the intrapersonal dimension (managing emotions, finding meaning) and the interpersonal dimension (facilitating social reintegration, enhancing communication skills) to break this cycle effectively.

### 4.5 Limitations and future directions

The interpretations of our findings must be considered in light of several limitations. Firstly, the cross-sectional design precludes definitive causal inference. While our model is theoretically grounded, longitudinal or interventional studies are required to confirm the direction of causality and the efficacy of support-focused interventions. Secondly, the use of a convenience sample from tertiary hospitals in one city may limit the generalizability of our findings to the broader IBD population in China. Thirdly, reliance on self-reported measures is susceptible to biases. Future research should incorporate objective clinical indices (e.g., disease activity scores, endoscopic findings) and physician-reported outcomes to triangulate findings. Furthermore, exploring these relationships across different disease phases (active vs. remission) and phenotypes (CD vs. UC) could yield more nuanced insights for personalized care. Finally, qualitative studies are highly recommended to delve deeper into the lived experiences of demoralization and the specific nature of support needs from the patients’ perspective, informing the development of more culturally sensitive and patient-centered interventions.

## 5 Conclusion

This study provides evidence that social support partially mediates the relationship between quality of life and demoralization in IBD patients. While causal inferences cannot be drawn, the findings suggest that enhancing social support may be a promising target for interventions aimed at reducing demoralization and improving quality of life. Future longitudinal and interventional studies are needed to confirm these pathways and evaluate the efficacy of psychosocial support programs in this population.

## Data Availability

The original contributions presented in the study are included in the article/supplementary material, further inquiries can be directed to the corresponding authors.
